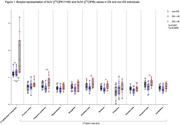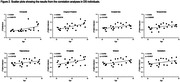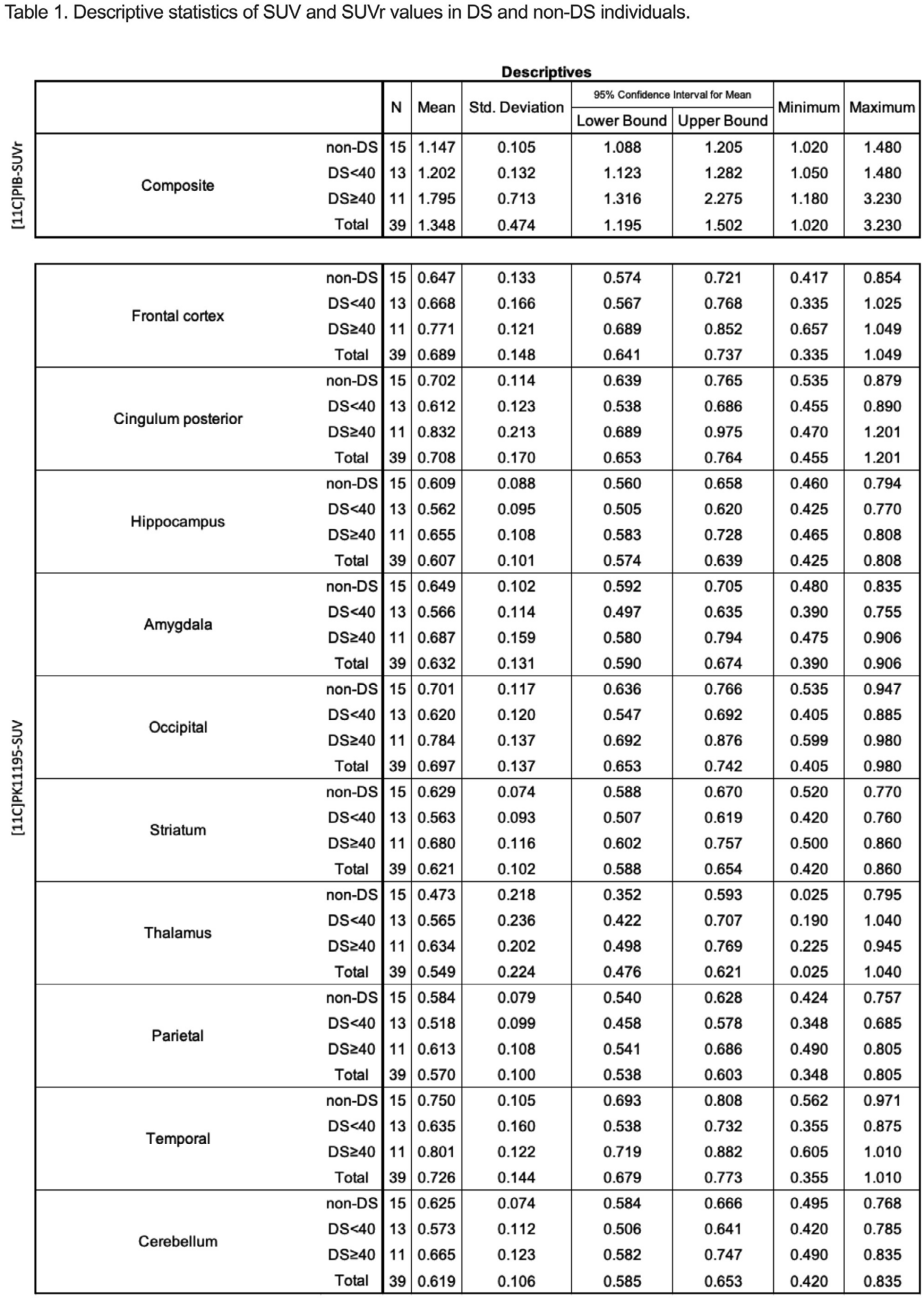# Neuroinflammation in people with Down syndrome and its association with amyloid deposition: a PET imaging study throughout aging

**DOI:** 10.1002/alz.090934

**Published:** 2025-01-09

**Authors:** Dimitri Brigide De Almeida Mantovani, Laura Cavalcanti de Oliveira, Artur Martins Coutinho, Ana Maria Marques da Silva, Orestes Vicente Forlenza, Carlos Alberto Buchpiguel, Daniele de Paula de Paula Faria

**Affiliations:** ^1^ University of São Paulo Medical School, São Paulo Brazil; ^2^ University of São Paulo Medical School, São Paulo, São Paulo Brazil

## Abstract

**Background:**

Down syndrome (DS) is a genetic disorder characterized by trisomy 21, which is linked to molecular alterations, such as neuroinflammation and accumulation of amyloid‐beta (Ab) peptide that can cause early‐onset Alzheimer's disease (AD). This study aims to evaluate the presence of neuroinflammation in individuals with DS in different ages and the association with Ab plaques.

**Methods:**

Age‐matched DS (n=24) and non‐DS individuals (n=15) underwent two hybrid PET/MRI acquisition for neuroinflammation and Ab burden assessment using [^11^C]PK11195 (∼370 MBq and 60 min acquisition) and [^11^C]PIB (∼370 MBq and 70 min acquisition) tracers, respectively. Quantification is presented as standard uptake values (SUV 40‐60min) for [^11^C]PK11195 (PMOD software) and SUVratio (SUVr 50‐70 min) for [^11^C]PIB using cerebellum grey matter as reference tissue (CortexID software) and cortical areas as a unique VOI called “composite”. Statistical analyses were performed dividing the DS group in <40 (n=13) and ≥40 (n=11) years of age. Differences in [^11^C]PK11195‐SUV and [^11^C]PIB‐SUVr were assessed by applying a multivariate one‐way ANOVA test with Bonferroni analysis for multiple comparisons. Correlation analyses were applied between [^11^C]PK11195‐SUV, [^11^C]PIB‐SUVr, and age variables.

**Results:**

Significant higher [11C]PK11195‐SUV was found in the occipital lobe (p<0.001), temporal lobe (p<0.001), and cingulum posterior (p<0.0001) when comparing the older to younger DS groups. Significant [^11^C]PIB‐SUVr differences were found for all groups, with the DS≥40 group showing the highest [11C]PIB uptake, followed by the DS<40 and non‐DS groups. No correlations were found between [^11^C]PK11195 and [^11^C]PIB measurements. However, for DS individuals, positive significant correlations with age was observed for [^11^C]PK11195‐SUV in the occipital lobe (p<0.05), temporal lobe (p<0.01), cingulum posterior (p<0.001), hippocampus (p<0.05), amygdala (p<0.05), striatum (p<0.05), and cerebellum (p<0.05), and for [11C]PIB‐SUVr in the composite VOI (p<0.01).

**Conclusion:**

[^11^C]PK11195‐PET analysis suggests increased neuroinflammation in older DS subjects. [^11^C]PIB‐PET results show a higher Ab burden in DS≥40 individuals and early accumulation of Ab in DS<40 individuals in comparison to non‐DS age‐matched subjects. More in‐vivo studies are needed to understand the temporal relationship between neuroinflammation and Ab deposition in the brain of individuals with DS.